# Supplementary material for: Osmotic Stress Leads to Significant Changes in Rice Root Metabolic Profiles between Tolerant and Sensitive Genotypes
Source: Plants (Basel). 2020 Nov 6;9(11):1503. doi: 10.3390/plants9111503 (PMC7694650; doi:10.3390/plants9111503)
Supplement: Supplementary file 1 [file plants-09-01503-s001.pdf]

Table S1. Top 50 and bottom loading of primary component (PC)1 and PC2 among 276 metabolites in the roots of two rice cultivars.

Ranking	PC1	Loding	PC2	Loding
1	β-Ala	0.1133	Mucic acid	0.1344
2	Hypotaurine	0.1127	Glucaric acid	0.1247
3	3-Aminoisobutyric acid	0.1116	Glucosaminic acid	0.1192
4	Thr	0.1097	Pyridoxine	0.1180
5	Pro	0.1075	Allantoin	0.1158
6	Ser	0.1062	N-Acetylglucosamine 1-phosphate	0.1090
7	O-Acetylserine	0.1051	Trimethylamine	0.1039
8	cGMP	0.1050	Trimethylamine N-oxide	0.1036
9	Hydroxyproline	0.1032	Betaine	0.1016
10	N6-Methyllysine	0.1029	Cysteinesulfinic acid	0.1009
11	CMP-N-acetylneuraminate	0.1029	N-Acetyl-β-alanine	0.0991
12	Glutathione (GSSG)_divalent	0.1020	Pyridoxal	0.0981
13	SDMA	0.1014	Vanillic acid	0.0980
14	Phe	0.1014	N-Acetylcysteine	0.0961
15	5-Aminovaleric acid	0.1014	Phosphoenolpyruvic acid	0.0952
16	Imidazole-4-acetic acid	0.1005	Sulfotyrosine	0.0927
17	His	0.1002	2-Hydroxyvaleric acid	0.0917
18	cCMP2',3'-cCMP	0.0991	UDP-N-acetylglucosamine	0.0875
19	Methionine sulfoxide	0.0988	Glyoxylic acid	0.0863
20	myo-Inositol 1-phosphate	0.0985	Glycolic acid	0.0861
21	Glucose 1-phosphate	0.0976	AMP	0.0857
22	Cysteine glutathione disulfide	0.0970	p-Hydroxymandelic acid	0.0856
23	Asp	0.0947	3',5'-ADP	0.0843
24	Glycerophosphocholine	0.0940	Ethyl glucuronide	0.0835
25	Tyr	0.0939	Carboxymethyllysine	0.0826
26	Lys	0.0938	Nicotinic acid	0.0810
27	Val	0.0937	N-Acetylglucosamine 6-phosphate	0.0799
28	Nω-Methylarginine	0.0935	Gluconic acid	0.0795
29	N6,N6,N6-Trimethyllysine	0.0929	XC0065	0.0794
30	Cadaverine	0.0923	2,4-Diaminobutyric acid	0.0761
31	γ-Glu-2-aminobutyric acid	0.0915	Cytosine	0.0756
32	3'-CMP	0.0914	Cysteic acid	0.0745
33	S-Methylmethionine	0.0908	Ethanolamine phosphate	0.0745
34	N-Acetylputrescine	0.0906	GMP	0.0741
35	Serotonin	0.0899	γ-Glu-2-aminobutyric acid	0.0739
36	Glu	0.0896	2,5-Dihydroxybenzoic acid	0.0728
37	Acetyl CoA_divalent	0.0890	XC0071	0.0728
38	N-Methylalanine	0.0890	S-Carboxymethylcysteine	0.0718
39	ADMA	0.0885	Ergothioneine	0.0696
40	N6-Acetyllysine	0.0879	Cystine	0.0687
41	Ophthalmic acid	0.0875	N-Formylglycine	0.0677
42	Thiaproline	0.0873	Mevalonic acid	0.0675
43	Octopamine	0.0868	3-Phosphoglyceric acid	0.0672
44	N-Acetylglutamine	0.0866	1,3-Diaminopropane	0.0667
45	1-Methyladenosine	0.0860	2,6-Diaminopimelic acid	0.0662
46	FAD_divalent	0.0859	2-Aminoadipic acid	0.0661
47	N-Acetylserine	0.0855	Ophthalmic acid	0.0648
48	N-Acetylornithine	0.0853	myo-Inositol 1-phosphatemyo-Inosi	0.0646
49	Saccharopine	0.0851	Ribulose 1,5-diphosphate	0.0643
50	Sucrose 6'-phosphate	0.0850	NMN	0.0642

Ranking	PC1	Loding	PC2	Loding
227	Sebacic acid	-0.0017	Pelargonic acid	-0.0668
228	Vanillic acid	-0.0020	CoA_divalent	-0.0672
229	Trimethylamine	-0.0021	Ornithine	-0.0676
230	Malic acid	-0.0029	Tyr	-0.0677
231	cis-Aconitic acid	-0.0030	Arg	-0.0680
232	Norspermidine	-0.0033	γ-Glu-Cys	-0.0685
233	GMP	-0.0038	Adenosine	-0.0699
234	2-Hydroxyvaleric acid	-0.0044	Citric acid	-0.0703
235	2-Oxoisovaleric acid	-0.0056	Allantoic acid	-0.0716
236	Uric acid	-0.0056	Glucose 6-phosphate	-0.0718
237	Adenine	-0.0071	Rhein	-0.0757
238	Adenosine	-0.0079	Homoserinelactone	-0.0761
239	Morpholine	-0.0082	cis-Aconitic acid	-0.0772
240	11-Aminoundecanoic acid	-0.0108	Adenine	-0.0774
241	1H-Imidazole-4-propionic acid	-0.0113	Guanosine	-0.0776
242	Threonic acid	-0.0134	Cytidine	-0.0777
243	AMP	-0.0135	S-Adenosylmethionine	-0.0801
244	CMP	-0.0166	Dyphylline	-0.0816
245	m-Hydroxybenzoic acid	-0.0173	GTP	-0.0826
246	3',5'-ADP	-0.0202	XA0002	-0.0852
247	2-Isopropylmalic acid	-0.0211	6-Phosphogluconic acid	-0.0856
248	Lauric acid	-0.0246	Spermidine	-0.0859
249	Pyridoxal	-0.0252	GDP-glucose	-0.0860
250	Octanoic acid	-0.0257	Quinic acid	-0.0871
251	2-Hydroxy-4-methylvaleric acid	-0.0272	3'-AMP	-0.0879
252	Shikimic acid	-0.0272	UDP-glucoseUDP-galactose	-0.0884
253	Trimethoprim	-0.0277	ATP	-0.0903
254	Glucuronic acid	-0.0311	Glucuronic acid	-0.0904
255	Guanine	-0.0315	2-Isopropylmalic acid	-0.0914
256	2,5-Dihydroxybenzoic acid	-0.0331	UTP	-0.0915
257	Gulonolactone	-0.0332	5-Amino-4-oxovaleric acid	-0.0915
258	Phosphorylcholine	-0.0337	Fructose 6-phosphate	-0.0919
259	NMN	-0.0338	Uridine	-0.0923
260	XA0033	-0.0362	Ribulose 5-phosphate	-0.0929
261	Cyclohexylamine	-0.0368	Malic acid	-0.0931
262	Gluconolactone	-0.0387	Succinic acid	-0.0951
263	3-Hydroxy-3-methylglutaric acid	-0.0425	Fumaric acid	-0.0953
264	2-Hydroxyglutaric acid	-0.0429	CTP	-0.0953
265	Succinic acid	-0.0430	Pantothenic acid	-0.0978
266	UMP	-0.0434	NADPH_divalent	-0.0997
267	Oxypurinol	-0.0438	N-Acetylglutamic acid	-0.1004
268	Gly-Asp	-0.0439	myo-Inositol 2-phosphate	-0.1025
269	5-Amino-4-oxovaleric acid	-0.0441	5'-Deoxy-5'-methylthioadenosine	-0.1029
270	Glutaric acid	-0.0448	Isocitric acid	-0.1033
271	Glycerol	-0.0555	Argininosuccinic acid	-0.1039
272	Trigonelline	-0.0582	Histidinol	-0.1041
273	Quinic acid	-0.0636	Citramalic acid	-0.1087
274	2-Oxoglutaric acid	-0.0766	Tyramine	-0.1102
275	Azetidine 2-carboxylic acid	-0.0785	XC0089	-0.1178
276	Terephthalic acid	-0.0841	UDP-glucuronic acid	-0.1186
